# Case of Elephantiasis Arabum of the Right Inferior Extremity, Successfully Treated by Ligature of the Femoral Artery

**Published:** 1853-09

**Authors:** J. M. Carnochan


### RECORD OF MEDICAL SCIENCE.

### SURGERY.

*Case of Elephantiasis Arabum of the Right Inferior Extremity, successfully treated by ligature of the Femoral Artery.* By J. M. CARNOCHAN, M. D.—Charles Roller, of lymphatic temperament, and short stature, æt. 27, born in Aix-la-Chapelle, occupation, merchant, left his home in December, 1849, landed in New York in February, 1851, went thence to Connecticut, where for eight months he worked in a factory, standing during his hours of labors; thence went to Virginia, where he worked on a farm for about six months, at the expiration of which period he was taken with fever, of an intermittent character. Up to that time, he had always been in good health.

During the fever, the inguinal glands became swollen and painful; the swelling and pain extending in the course of the femoral vessels as far as the knee. The pain was followed by swelling and redness of the thigh down to the knee. From the knee, the pain and swelling continued to extend downwards as far as the toes; being, at this time confined chiefly to the portions of the limb along the course of the saphena vein, and also of the posterior tibial vessels. The redness and tumefaction here, as in the thigh, were preceded by deep-seated pain. The tumefaction of the limb continued to increase; while, at the same time, febrile exacerbations occurred at intervals, varying from two to six days. After a period of about six weeks from the commencement of the disease, the fever entirely disappeared, and by this time, also, the pain and redness had entirely ceased; the limb, however, remaining hard, swollen and rough, and presenting, in a marked degree, the peculiar characteristics of Elephantiasis Arabum, in the chronic period of the disease. From this time forward, the hardness and intumescence gradually increased, and the limb became so cumbersome that the patient was obliged to give up all business, and confine himself chiefly to a recumbent posture. In this condition, the patient left Virginia for the purpose of seeking medical relief at the New York Emigrants' Hospital, into which he was admitted the fifteenth of January, 1851. The appearance of the patient upon entering the Hospital was somewhat emaciated. He had no febrile symptoms, and the chief difficulty, under which he labored, arose from the enlarged and hypertrophied condition of the right inferior extremity.

The limb was enlarged from the toes to within a short distance below Poupart's ligament. The thigh, although enlarged, was not much indurated; while, from a short distance above the patella, downwards, the limb presented a dense, hypertrophied, hard, scaly, shapeless mass.

The morbid condition of the tissues pervaded the foot and toes, there presenting groups of tuberculated growths. The circumference of the limb around the ankle, was nearly as large as that of the calf; measuring fifteen and one-half inches, while the circumference of the calf measured nineteen and one half inches.

The patient was put under treatment upon entering the Hospital. The recumbent posture was enjoined, and for some time various discutient lotions were used. Bandaging was resorted to, with frictions of ung. Potass. Iodid.; the Iodide of Potassium being also prescribed internally.

At times, also, the limb was painted with strong tincture of Iodine; local and general baths were used, regular bandaging of the limb, from the toes upward, being the while carefully observed.

This plan of treatment was perseveringly adhered to from the fifteenth of January to the twenty-second of March, a period of a little over two months, without any amelioration. Having thus tried, without success, the method of treatment most approved of, I proposed to place a ligature upon the femoral artery, with a view of changing the morbid condition of the structures supplied by the branches of this arterial trunk. A consultation was held, and my proposition was acceded to as preferable to amputation, the usual alternative resorted to in this stage and extent of the disease. Accordingly, on the twenty-second of March, 1851, I secured the femoral artery, a short distance below the origin of the arteria profunda. Upon exposing the femoral artery, this arterial tube was found to be changed, so as to present an appearance somewhat like the color of the aorta of the ox, and to be larger than the common iliac of the human subject. In consequence of this appearance of the artery, after some hesitation, I applied the ligature, preferring to do this, rather than to expose the external iliac, of the soundness of which I could not be certain.

The ligature came away from the femoral artery on the eleventh day, accompanied by secondary hemorrhage, the occurrence of which I expected as probable. For the purpose of arresting the hemorrhage, the *external* iliac artery was secured by ligature, by Dr. A. E. Hosack, who happened to be on duty at the time in the Hospital. The external iliac was found to be about the size of the brachial artery. This, for a time, apparently had some influence upon the hemorrhage; but on the following day, bleeding was again renewed from the orifice, in the femoral artery, with as much profusion as ever.

The hemorrhage was now restrained by the prompt application of a tourniquet, on the *cardiac* side of the bleeding orifice, by the house surgeons, Drs. Thompson and A. K. Smith.

This even failed to stop permanently the hemorrhage, and the blood recommenced oozing copiously at intervals. The patient was now sinking fast, and the ligature of the common iliac, or amputation at the hip-joiut, appeared to be the only resources left. But the hemorrhage now baing evidently reflux, it was suggested to apply the tourniquet, so as to produce compression on the *distal* side of the bleeding orifice: this was done, and was followed by a complete cessation of the bleeding.

From this time, (April fourth, 1851,) the house surgeon kept an instructive record of the case, which record I have now before me. For several days, the pulse ranged from 115 to 108 : the dressings were carefully attended to, and light diet prescribed. On the twelfth, the leg was found to be considerably reduced in size, and the ligature of the external iliac, came away. On the seventeenth, brandy and quinine, with good nourishment, were ordered. On May the first, finding the leg still more reduced and the lower wound healed, I ordered tincture of iodine to be painted on the leg, and the bandage to be continued; I also ordered a solution of chloride of soda to be used as a wash on the upper wound, which continued to discharge freely.

The patient now went on gradually improving in strength and appearance, and left the Hospital in the latter part of June, completely cured of his malady. At this date, sixteen months after the ligature of the femoral artery, the patient is in robust health, and presents no indications that the disease will return.

*Report of Dr. Carnochan.*

